# Abnormal Intracellular Accumulation and Extracellular Aβ Deposition in Idiopathic and Dup15q11.2-q13 Autism Spectrum Disorders

**DOI:** 10.1371/journal.pone.0035414

**Published:** 2012-05-02

**Authors:** Jerzy Wegiel, Janusz Frackowiak, Bozena Mazur-Kolecka, N. Carolyn Schanen, Edwin H. Cook, Marian Sigman, W. Ted Brown, Izabela Kuchna, Jarek Wegiel, Krzysztof Nowicki, Humi Imaki, Shuang Yong Ma, Abha Chauhan, Ved Chauhan, David L. Miller, Pankaj D. Mehta, Michael Flory, Ira L. Cohen, Eric London, Barry Reisberg, Mony J. de Leon, Thomas Wisniewski

**Affiliations:** 1 Department of Developmental Neurobiology, NYS Institute for Basic Research in Developmental Disabilities, Staten Island, New York, United States of America; 2 Nemours Biomedical Research, duPont Hospital for Children, Wilmington, Delaware, United States of America; 3 Department of Psychiatry, University of Illinois at Chicago, Chicago, Illinois, United States of America; 4 Department of Psychiatry, University of California Los Angeles, Los Angeles, California, United States of America; 5 Department of Human Genetics, NYS Institute for Basic Research in Developmental Disabilities, Staten Island, New York, United States of America; 6 Department of Neurochemistry, NYS Institute for Basic Research in Developmental Disabilities, Staten Island, New York, United States of America; 7 Department of Molecular Biology, NYS Institute for Basic Research in Developmental Disabilities, Staten Island, New York, United States of America; 8 Department of Psychology, NYS Institute for Basic Research in Developmental Disabilities, Staten Island, New York, United States of America; 9 Departments of Neurology, Pathology and Psychiatry, New York University School of Medicine, New York, New York, United States of America; University of Florida, United States of America

## Abstract

**Background:**

It has been shown that amyloid ß (Aβ), a product of proteolytic cleavage of the amyloid β precursor protein (APP), accumulates in neuronal cytoplasm in non-affected individuals in a cell type–specific amount.

**Methodology/Principal Findings:**

In the present study, we found that the percentage of amyloid-positive neurons increases in subjects diagnosed with idiopathic autism and subjects diagnosed with duplication 15q11.2-q13 (dup15) and autism spectrum disorder (ASD). In spite of interindividual differences within each examined group, levels of intraneuronal Aβ load were significantly greater in the dup(15) autism group than in either the control or the idiopathic autism group in 11 of 12 examined regions (p<0.0001 for all comparisons; Kruskall-Wallis test). In eight regions, intraneuronal Aβ load differed significantly between idiopathic autism and control groups (p<0.0001). The intraneuronal Aβ was mainly N-terminally truncated. Increased intraneuronal accumulation of Aβ_17–40/42_ in children and adults suggests a life-long enhancement of APP processing with α-secretase in autistic subjects. Aβ accumulation in neuronal endosomes, autophagic vacuoles, Lamp1-positive lysosomes and lipofuscin, as revealed by confocal microscopy, indicates that products of enhanced α-secretase processing accumulate in organelles involved in proteolysis and storage of metabolic remnants. Diffuse plaques containing Aβ_1–40/42_ detected in three subjects with ASD, 39 to 52 years of age, suggest that there is an age-associated risk of alterations of APP processing with an intraneuronal accumulation of a short form of Aβ and an extracellular deposition of full-length Aβ in nonfibrillar plaques.

**Conclusions/Significance:**

The higher prevalence of excessive Aβ accumulation in neurons in individuals with early onset of intractable seizures, and with a high risk of sudden unexpected death in epilepsy in autistic subjects with dup(15) compared to subjects with idiopathic ASD, supports the concept of mechanistic and functional links between autism, epilepsy and alterations of APP processing leading to neuronal and astrocytic Aβ accumulation and diffuse plaque formation.

## Introduction

Autism is a developmental disorder characterized by qualitative impairments in reciprocal social interactions, verbal and nonverbal communication, and restricted, repetitive and stereotyped patterns of behavior [1]. Autism is often diagnosed in subjects with genetic disorders, including maternal origin duplications 15q11.2-q13 (dup15) (69%) [2], [3], fragile X syndrome (FXS) (15–28%) [4] and Down syndrome (DS) (7%) [5].

Recent studies indicate that non-amyloidogenic cleavage of the amyloid-β (Aβ) peptide precursor (APP) with α and γ secretases is linked to several developmental disorders, including autism and FXS [6]–[10]. The proteolytic cleavage of APP by membrane-associated secretases releases several Aβ peptides possessing heterogeneous amino- and carboxyl-terminal residues, including Aβ_1–40_ and Aβ_1–42_ as products of β- and γ-secretases (amyloidogenic pathway); Aβ_17–40/42_, as a product of α- and γ-secretases (p3 peptide, non-amyloidogenic pathway) [11], [12]; and Aβ_pE3_ as a product of N-terminal truncation of full-length Aβ peptide by aminopeptidase A and pyroglutamate modification [13]. Aβ peptides differ in toxicity, oligomerization, fibrillization, distribution and trafficking within cells, and in their contribution to Aβ deposits in plaques and vascular walls. Alzheimer disease (AD) is associated with oligomeric Aβ accumulation, fibrillar Aβ deposition in plaques, neuronal degeneration and cognitive decline. Intraneuronal Aβ accumulation has been shown to be an early event in AD brains and in transgenic mouse models of AD, and has been linked to synaptic pathology [14], [15].

Detection of significantly increased levels of sAPP-α in blood plasma in 60% of autistic children was reported to be an early biomarker of a subgroup of children with autism [6]. Enhanced APP processing by α-secretase is especially prominent in autistic subjects with aggressive behavior [6], [16]. Sokol et al. [10] proposed that increased levels of sAPP-α contribute to both the autistic and FXS phenotypes, and that excessively expressed sAPP-α neurotrophic activity may contribute to an abnormal acceleration of brain growth in autistic children and to macrocephaly in FXS. The fragile X mental retardation protein (FMRP) binds to and represses the dendritic translation of APP mRNA, and the absence of FMRP in FXS and in *Fmr1^KO^* mice results in the upregulation of APP, Aβ_40_ and Aβ_42_ [7]. Westmark et al. [8] also revealed that genetic reduction of *AβPP* by removal of one *App* allele in *Fmr1^KO^* mice results in reversion of FXS phenotypes, including reduction of plasma Aβ_1–42_, to normal levels. Experimental studies in *Fmr1^KO^* mice [17] suggest that over-expression of APP/Aβ may contribute to the seizures observed in autism [18] and FXS [4] and that both the over- and under-expression of APP and its metabolites increase the incidence of seizures [7], [17], [19], [20].

Previously we reported that in the brains of controls, both children and adults, neurons accumulate cell type–specific amounts of Aβ_17–40/42_, which is the product of nonamyloidogenic APP processing [21]. One may hypothesize that increased levels of sAPP-α in blood plasma [6], [9], [16] reflect the enhanced non-amyloidogenic processing of neuronal APP with α-secretase in the brain of autistic subjects.

The aims of this comparative study of the brains of subjects with idiopathic autism (autism of unknown etiology) and autism caused by maternal origin dup(15) were (a) to test the hypothesis that regardless of the causative mechanism, autism is associated with an enhanced accumulation of Aβ in neuronal cytoplasm, (b) to show that intraneuronal Aβ is the product of non-amyloidogenic α-secretase APP cleavage (Aβ_17–40/42_), (c) to show brain region– and cell type–specific Aβ immunoreactivity, and (d) to identify cytoplasmic organelles involved in Aβ accumulation in the neurons of autistic and control subjects.

## Results

### The Difference between Intraneuronal Aβ Accumulation in dup(15) Autism, Idiopathic Autism and Control Groups

**Figure 1:**
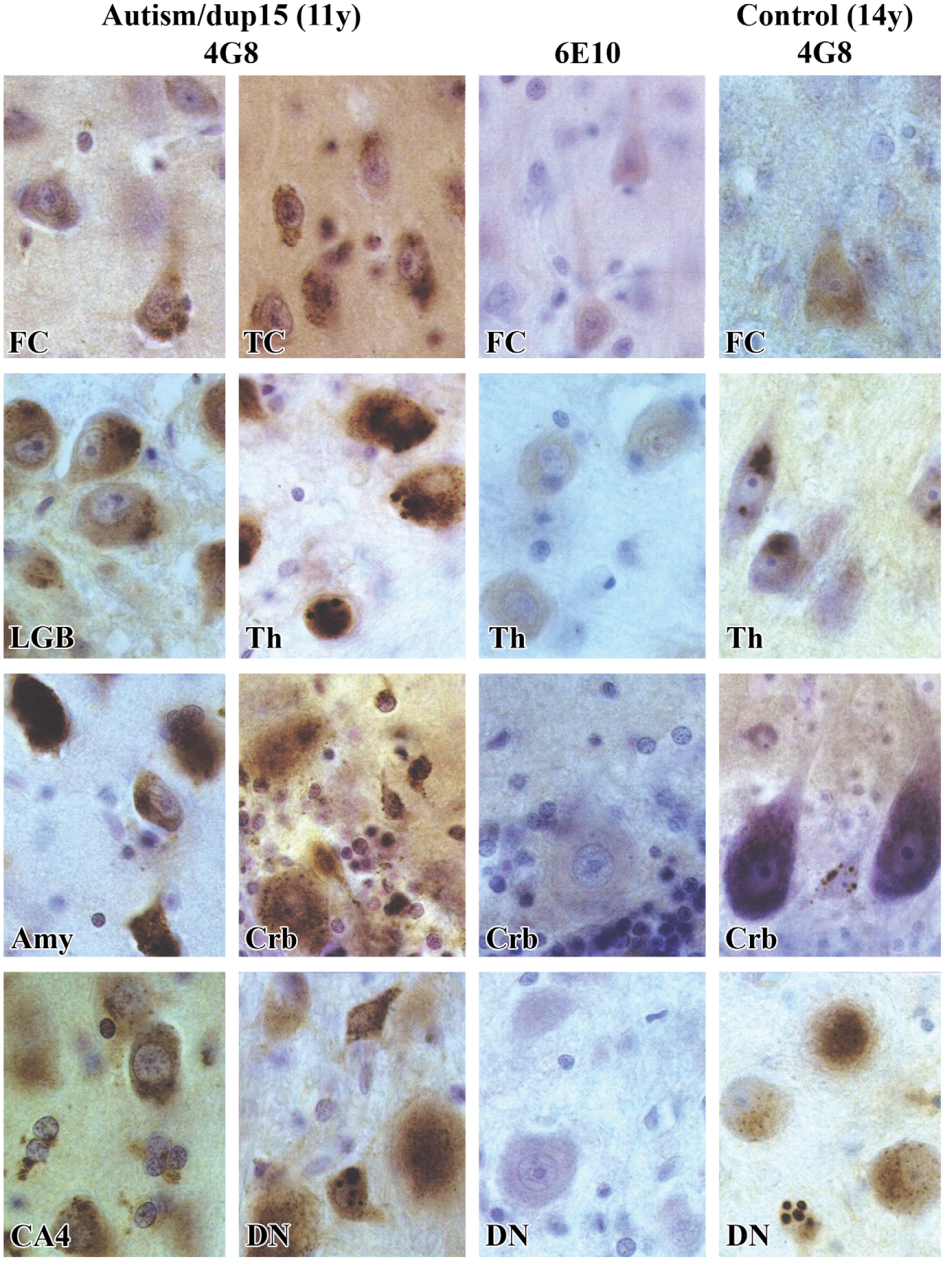
Enhanced intraneuronal accumulation of amino-terminally truncated Aβ in autism. Mapping of Aβ_17–24_ in the brain AN09402 reveals brain region– and cell type–specific patterns of abnormal Aβ accumulation in the cytoplasm of neurons and glial cells of a male diagnosed with dup(15), autism and intractable epilepsy, whose sudden unexpected death at the age of 11 years was seizure-related. Almost all neurons in the frontal (FC) and temporal cortex (TC) are 4G8-positive, but the reaction intensity varies from weak to strong. Strong immunoreactivity is observed in many neurons in the lateral geniculate body (LGB), thalamus (Th), amygdala (Amy), Purkinje neurons and basket and stellate neurons in the molecular layer in the cerebellar (Crb) cortex, in many neurons and astrocytes in the CA4, and large and small neurons in the dentate nucleus (DN). Some types of neurons (in the reticular nucleus in the thalamus and small neurons in the dentate nucleus) have different types of deposits: fine-granular and 2- to 3-µm in diameter 4G8-positive deposits. No reaction or only traces of a reaction detected with mAb 6E10 in the frontal cortex, thalamus, cerebellum and dentate nucleus indicate that in intraneuronal Aβ the amino-terminal portion is missing, and the prevalent form of Aβ is α-secretase product. Immunoreactivity with mAb 4G8 is present in the brain of the control subject (14 years of age), but fewer neurons are positive, and immunoreactivity in the frontal cortex, thalamus, cerebellum and dentate nucleus is weaker than in the affected subject. In the control subject, glial cells are usually 4G8-immunonegative.

In all subjects with dup15/autism spectrum disorder (ASD) and the majority of individuals with idiopathic ASD, intraneuronal Aβ immunoreactivity was observed in more neurons, and the amount of immunoreactive material was increased in comparison to the control subjects (Fig. 1). The morphology of the intracellular deposits of Aβ-positive material was cell type–specific. Cortical pyramidal neurons showed significant heterogeneity of intraneuronal deposits with a mixture of fine granular material and several times larger 4G8-positive granules. In Purkinje cells, fine granular deposits were accumulated in the cell body. In the dentate nucleus, large neurons accumulated fine granular material, whereas small neurons accumulated a few much larger Aβ-positive granules. Neurons in the reticulate nucleus in the thalamus contained a mixture of fine granular material and large 4G8-positive granules.

Immunocytochemistry with monoclonal antibodies (mAbs) 4G8 (17–24 aa of Aβ) and 6E10 (4–13 aa of Aβ) revealed that almost all intraneuronal Aβ is 4G8-positive, but only a very small proportion is labeled with 6E10.

Quantitative evaluation of 12 brain subregions/cell types (frontal, temporal and occipital cortex, Purkinje cells, amygdala, thalamus, lateral geniculate body (LGB), dentate gyrus, CA1 and CA4 sectors and dentate nucleus) revealed that in 11 subregions intraneuronal Aβ load was significantly greater in the dup(15) autism group than in the control and idiopathic autism cohorts (p<0.0001 for all comparisons). In eight regions (all three cortical subregions, Purkinje cells, amygdala, thalamus, LGB, and dentate gyrus), intraneuronal Aβ load differed significantly between the idiopathic autism and control groups (p<0.0001). In structures with almost all neurons positive for Aβ–the dentate nucleus and the inferior olive–the amyloid load was insignificantly higher in control subjects than in subjects with idiopathic autism.

**Figure 2:**
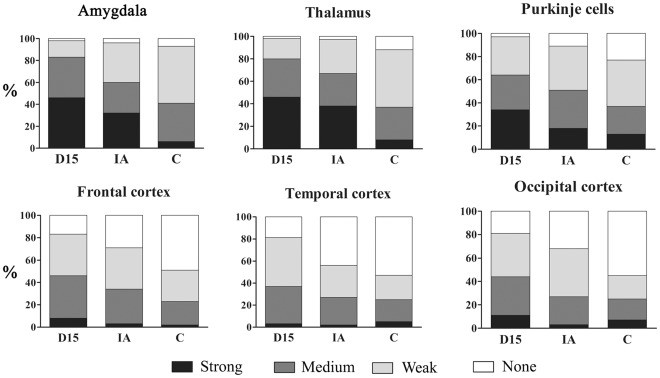
Two major patterns of alterations in intraneuronal Aβ accumulation. Graphs show a high percentage of neurons with strong cytoplasmic immunoreactivity (mAb 4G8) in the amygdala, thalamus and Purkinje cells in subjects diagnosed with dup(15) autism (D15), a lower percentage in idiopathic autism (IA) subjects, and a low percentage in control subjects. In contrast, the characteristic feature of pyramidal neurons in the frontal, temporal and occipital cortex is a low percentage of neurons with strong Aβ immunoreactivity, whereas the total percentage of Aβ-positive neurons is significantly higher in the dup(15) group than in the idiopathic autism group or in control subjects. Differences in Aβ immunoreactivity in the dup(15) autism vs. control cohort, the idiopathic autism vs. control group, and the dup(15) autism vs. idiopathic autism are significant (p<0.0001).

Quantitative study revealed different patterns of immunoreactivity in brain subregions (Fig. 2, and Supporting Information, Fig. S1). The characteristic feature distinguishing the amygdala, thalamus and Purkinje cells of subjects with dup(15) autism was the very high percentage of neurons with strong cytoplasmic Aβ immunoreactivity (46%, 46% and 35%, respectively); the percentage was significantly lower in the idiopathic autism group (32%, 38% and 19%, respectively), and very low in control subjects (6%, 6% and 12%, respectively). However, in pyramidal neurons in the frontal, temporal and occipital cortex, the percentage of neurons with strong Aβ immunoreactivity was low (3–10%), whereas the total percentage of Aβ-positive neurons was significantly higher in the dup(15) group (81–83%) than in the idiopathic autism group (56–71%) and in control subjects (45–51%).

The percentage of Aβ-positive neurons and neuronal amyloid load was smaller in the hippocampal formation, especially in the CA1 sector and dentate gyrus of control subjects. The amyloid load was significantly higher in the dup(15) autism group than in control subjects, but the difference in amyloid load between the idiopathic autism and control groups was insignificant (Fig. S1).

The feature distinguishing the LGB, inferior olive and dentate nucleus from other brain structures is the childhood onset of lipofuscin accumulation. In LGB, strong Aβ immunoreactivity was observed in 73% of neurons in dup(15) autism and in 62% in idiopathic autism but only 16% of LGB neurons were strongly Aβ-positive in control subjects. In the dentate nucleus, the percentage of strongly positive neurons was comparable in all three groups (41%, 35% and 41%, respectively), but overall amyloid load was statistically higher in dup(15) autism. The percentage of strongly Aβ-positive neurons in the inferior olive was the same in the idiopathic autism and in the dup(15) (32%) group, and there was no difference in overall amyloid load between autistic and control subjects (Fig. S1).

### Aβ in Glial Cells

**Figure 3:**
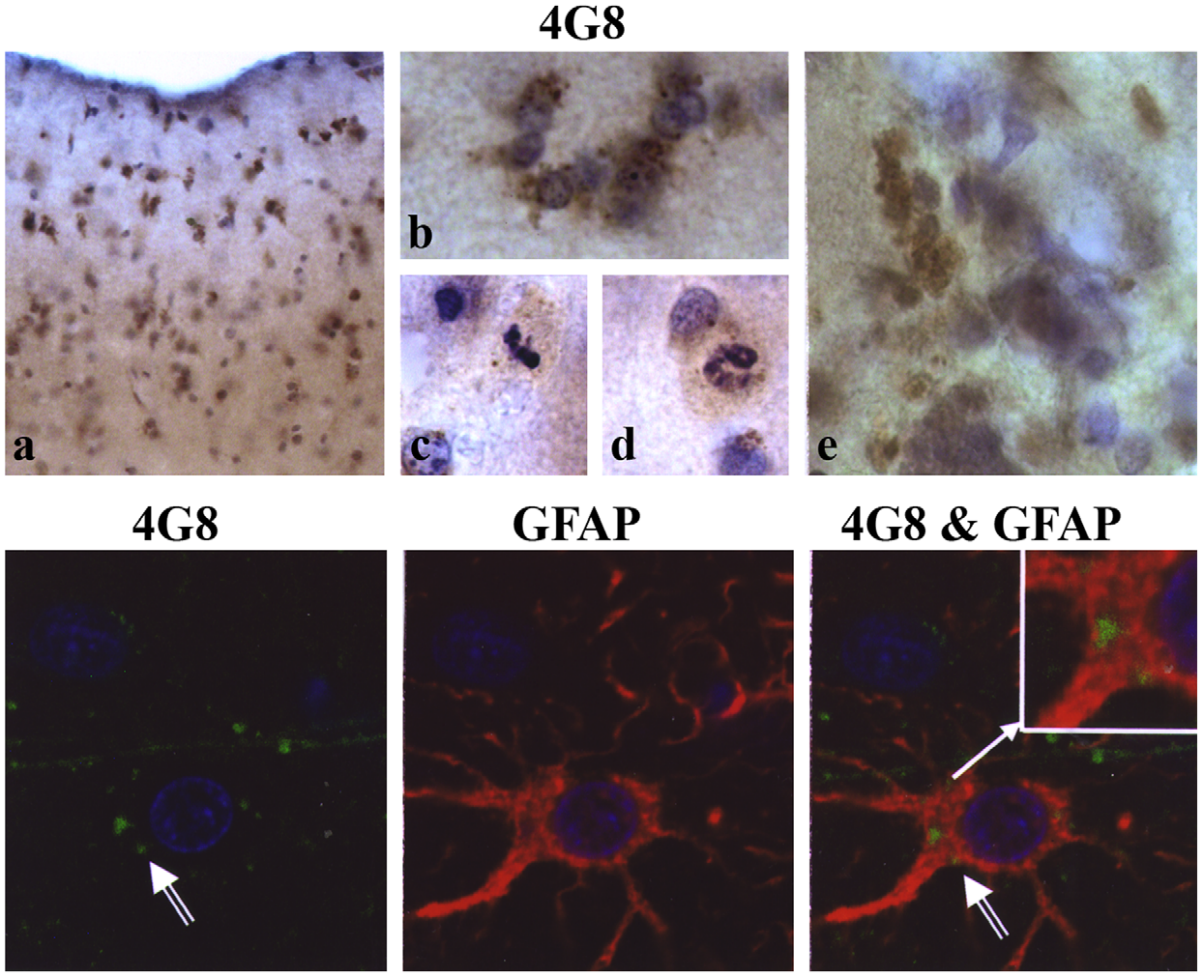
Enhanced accumulation of amino-terminally truncated Aβ in autistic subjects astrocytes. Clusters of 4G8-positive astrocytes, especially numerous in the molecular layer (a, b); very frequent mitotic divisions (c, d); and extracellular 4G8-positive Aβ deposits, with morphology of astrocytes’ cytoplasmic aggregates (e) may reflect the enhanced proliferation, degeneration and death of Aβ-positive astrocytes in the brain of autistic subjects. Confocal microscopy confirmed the presence of Aβ (green; arrows) in the cytoplasm of GFAP-positive astrocytes (red). Cell nuclei were stained with TO-PRO-3-iodide (blue).

Astrocytes and microglia in the control brains were usually Aβ-negative or contained only traces of Aβ immunoreactivity. Enhanced neuronal Aβ accumulation in the brains of individuals with autism was associated with Aβ accumulation in the astrocytes’ cytoplasm and in some microglial cells (Fig. 3). Two patterns of Aβ immunoreactivity were observed in astroglia. The most common form was a condensed aggregate of Aβ in one pole of the astrocyte soma typical for CA4 sector, some cortical areas but without clear anatomical predilection, and the cerebellar cortex border zone between granule and molecular layers. The less common form was deposition of Aβ-immunoreactive granular material in the entire astrocyte body and in a proximal portion of processes radiating from the cell body (frequent in the molecular layer of the cerebral cortex). The increase in the amount of cytoplasmic Aβ was often paralleled by (a) a several-fold increase in the number of astrocytes, all of which were Aβ-positive (Fig. 3a), (b) clustering of astrocytes in groups of 3–10 cells (Fig. 3b), (c) numerous mitoses as a sign of astrocyte proliferation (Fig. 3c,d) and (d) astrocyte death resulting in deposition of extracellular remnants of Aβ aggregates (Fig. 3e) similar to those seen in astrocyte cytoplasm. Extracellular Aβ deposits were found in neuropil, but larger aggregates (more than 10) were more often in the perivascular space. Confocal microscopy confirmed Aβ accumulation in GFAP-positive astrocytes (Fig. 3, lower panel).

### Intracellular Distribution of Amino-terminally Truncated Aβ in Neurons

**Figure 4:**
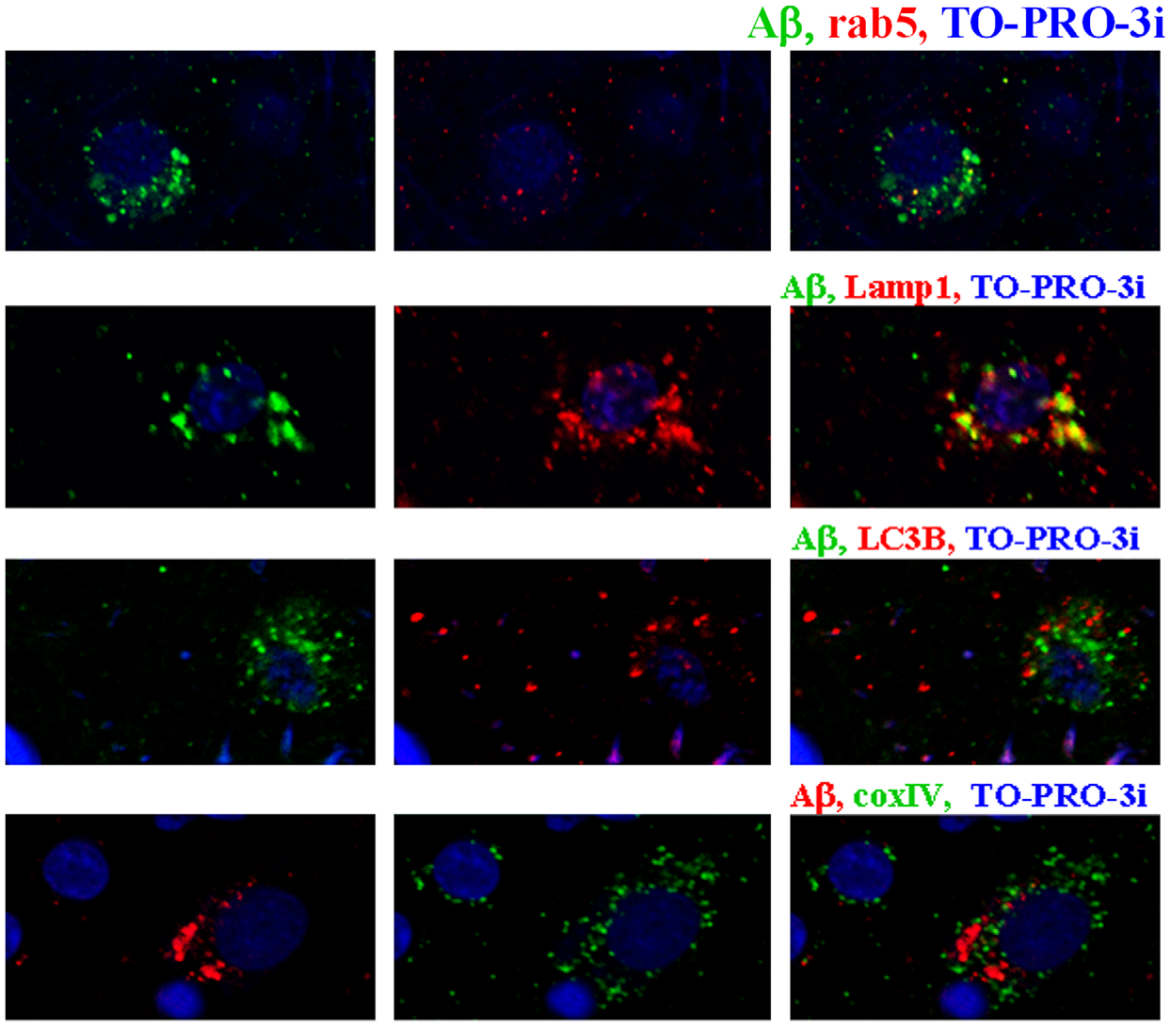
Aβ in endocytic vesicles, autophagic vacuoles, lysosomes and mitochondria. Co-localization of Aβ (4G8) in neurons in the frontal cortex of a 10-year-old subject diagnosed with autism/dup(15) (AN06365) demonstrates that a small portion of cytoplasmic Aβ is stored in rab5-positive endocytic vesicles and LC3B-positive autophagic vacuoles, whereas the largest proportion of Aβ is colocalized with lysosomal Lamp1. Colocalization of a relatively large portion of cytoplasmic Aβ with lysosomal markers appears to reflect the accumulation of products of intracellular degradation of Aβ that originated from endocytic and autophagic pathways. The presence of only a few Aβ-positive mitochondria immunolabeled with COXIV may suggest that this Aβ makes the smallest contribution to the detected neuronal Aβ accumulation and degradation pathway.

Intraneuronal Aβ deposits revealed striking neuron type–specific differences in amount, morphology and cytoplasmic distribution; however, they had the same immunoproperties. They revealed no reaction or traces of reaction with mAb 6E10 (Fig. 1) or 6F3D (not shown). The morphological diversity of Aβ deposits suggested that Aβ was present in different compartments of the endosomal-lysosomal pathway and in lipofuscin in neuron type–specific amounts. The number and size of Lamp1– (Fig. 4) lysosomes was from 2 to 3 times more than the number of Aβ-positive deposits; however, only about 10% of Aβ was detected in rab5-positive endosomal vesicles and in LC3B-positive autophagic vacuoles. Colocalization of Aβ with COXIV-positive mitochondria was observed in only a very few mitochondria.

**Figure 5:**
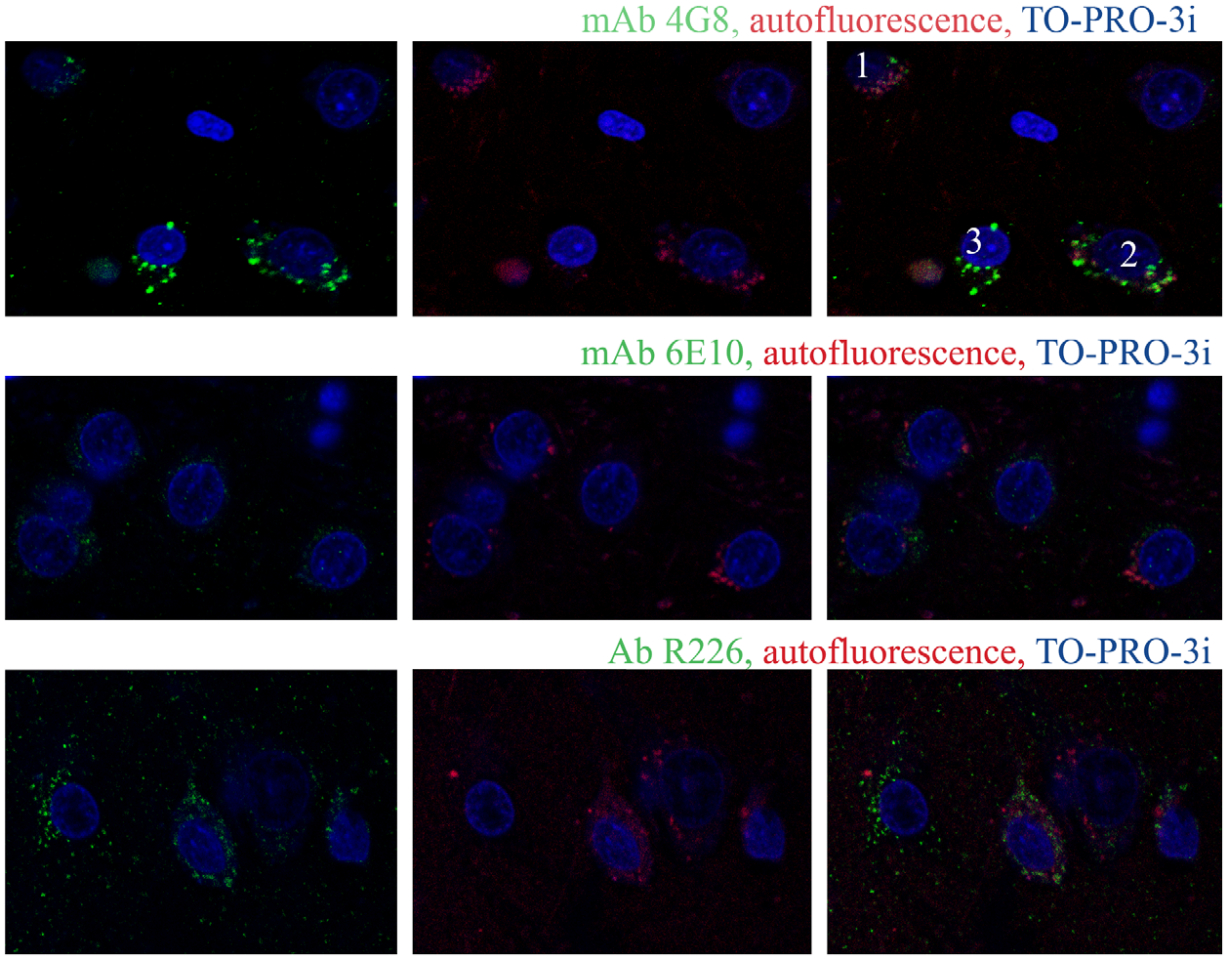
Aβ in lipofuscin. Accumulation of Aβ in lipofuscin in the frontal cortex of a 10-year-old subject diagnosed with dup(15) autism was characterized using mAbs 4G8 and 6E10 and pAb R226. Autofluorescent lipofuscin granules were 4G8-negative (cell 1) or partially positive (cell 2), but Aβ was also accumulated in lipofuscin-free neurons (cell 3). The neurons revealed only traces of reaction with mAb 6E10 and a moderate amount of pAb R226–positive Aβ42, which was partially co-localized with autofluorescent lipofuscin.

Immunoreaction for Aβ detected with mAb 4G8 was present in some intracellular autofluorescent granules; however, the 4G8-immunoreactive deposits were detected also in neurons with scanty lipofuscin (Fig. 5) and in neurons with abundant autofluorescent granules. On the other hand, some neurons with scanty immunoreaction for Aβ contained numerous autofluorescent granules. The autofluorescent granules were not immunostained with mAb 6E10. Immunoreaction with polyclonal antibody (pAb) R226, specific for the C-terminus of Aβ42, showed only a fraction of labeling colocalized with autofluorescent granules. These results indicate that the detected intraneuronal immunostaining reflects accumulation of N-terminally truncated Aβ in several cellular compartments, including lipofuscin granules.

### Specificity of Immunohistochemical Detection of Aβ with mAb 4G8 and 6E10

**Figure 6:**
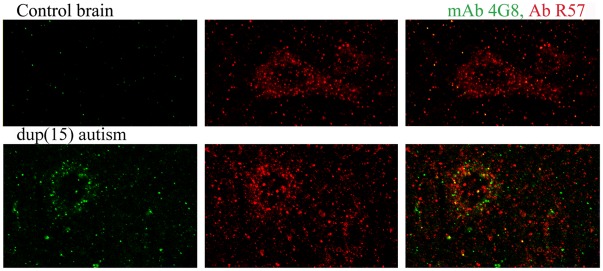
Immunoreactivity of mAb 4G8 with Aβ. mAb4G8 detects Aβ but does not detect APP in immunohistochemical staining in formalin-fixed and PEG-embedded samples of the frontal cortex of an 8-year-old control subject and a 10-year-old subject diagnosed with dup(15) and autism. Neurons in the control brain contain numerous granules that are immunoreactive with C-terminal APP–specific pAb R57 and are 4G8 negative. In the neurons of an autistic subject, only a few very numerous 4G8-positive deposits are R57-positive, whereas the majority of very numerous APP-immunoreactive granules are 4G8-negative.

The epitopes of mAbs 6E10 and 4G8 (4–13 aa and 17–24 aa of the Aβ sequence, respectively) are present in full-length APP and APP C-terminal fragments. In brain tissue that has been fixed in formalin for several months, embedded in polyethylene glycol (PEG) and pretreated with 70% formic acid for 20 min, the immunostaining with mAb 4G8 (Fig. 6) and with 6E10 and 7F3D (8–17 aa of Aβ; not shown) is consistent with the distribution and amount of Aβ, but different from the distribution and amount of neuronal APP. In control brains, antibody R57 detects abundant intraneuronal APP immunoreactivity, but mAb 4G8 reveals only a very limited reaction with Aβ. In numerous neuronal populations in autistic subjects, the immunoreactivity for Aβ increases very significantly, but most R57 immunoreactive material is 4G8-negative, and most 4G8-positive granules are negative for APP. These results indicate that in the examined material, mAbs 6E10, 4G8 and 7F3D detect Aβ but do not bind to neuronal APP detected with pAb R57.

### Diffuse Plaque Distribution and Immunoproperties in the Brain of Autistic Subjects

**Figure 7:**
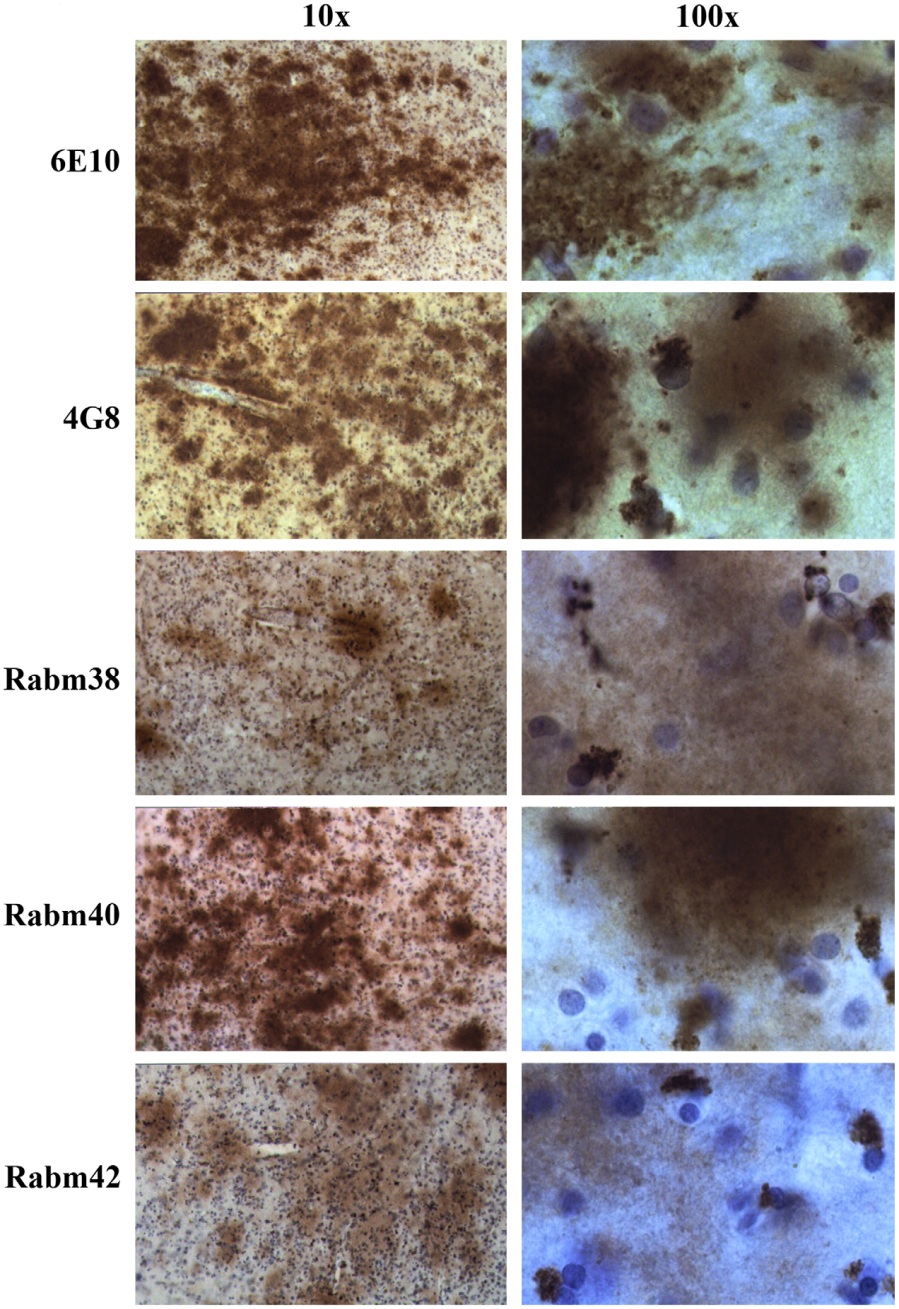
Full-length Aβ in diffuse plaques and amino-terminally truncated Aβ in astrocytes in autism/dup15. Diffuse plaques in the frontal cortex of a 39-year-old female (AN11931) diagnosed with dup(15), autistic features, and intractable seizures (age of onset 9 years) and whose death was epilepsy-related, are 6E10-, 4G8-, Rabm38-, Rabm40- and Rabm42-positive. Reaction with Rabm38 and Rabm42 was weaker than with other antibodies. Almost all glial cells with the morphology of astrocytes detected in the plaque perimeter had a large cluster of granular material located usually at one cell pole and positive with all antibodies detecting Aβ, except 6E10.

**Figure 8:**
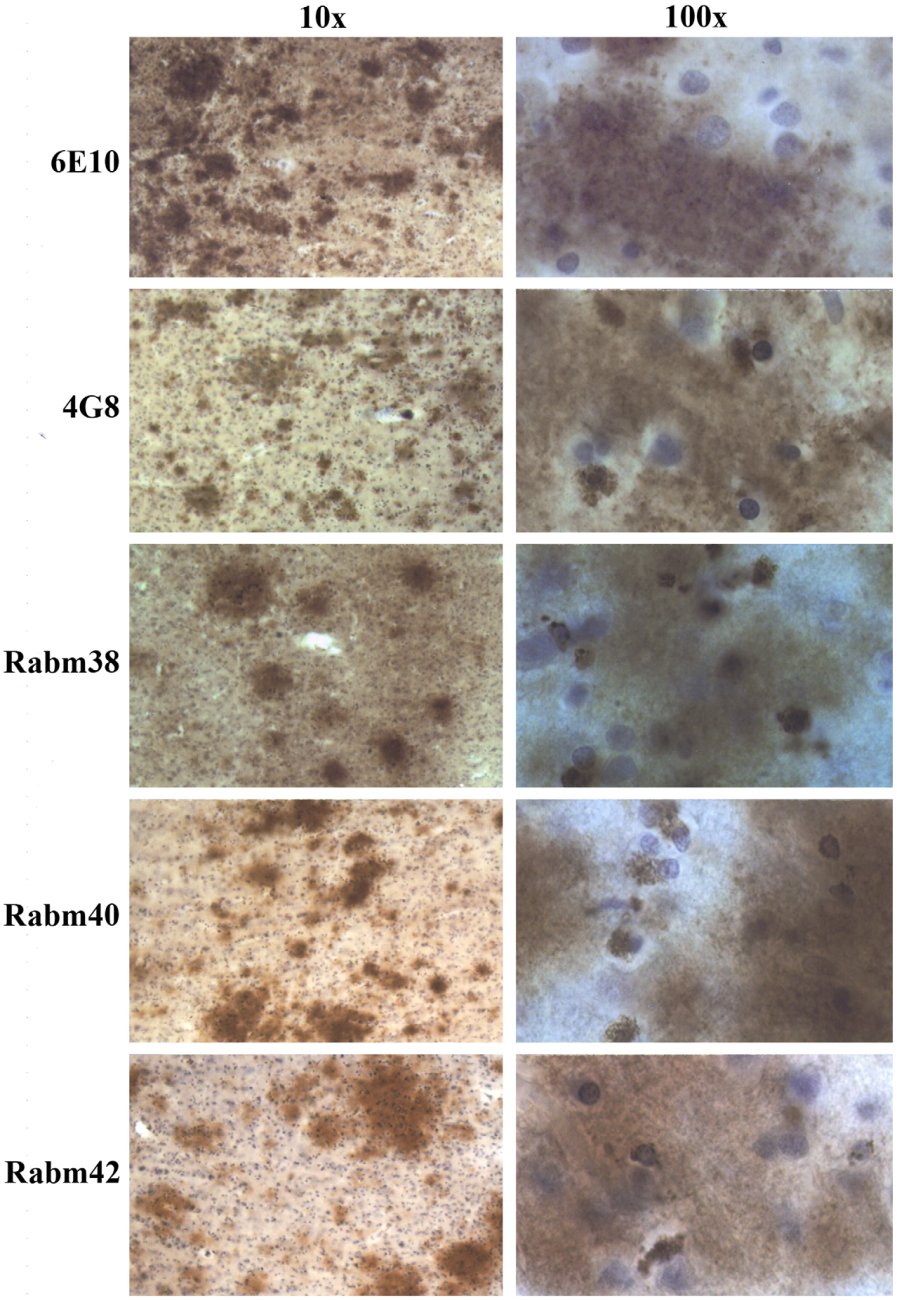
Full-length Aβ in diffuse plaques, and truncated Aβ in astrocytes in idiopathic autism. Diffuse plaques in the frontal cortex of a 51-year-old subject (AN17254) diagnosed with idiopathic autism, who had had only one grand mal seizure and died because of cardiac arrest, are immunopositive when stained with all five antibodies (6E10, 4G8, Rabm38, Rabm 40 and Rabm 42), but granular material in the cytoplasm of glial cells is immunopositive for all antibodies used except 6E10.

Aβ-positive plaques were detected in one of the nine examined subjects diagnosed with dup15 (AN11931), and in two of the 11 subjects diagnosed with idiopathic autism (AN17254 and BB1376). All three subjects were the oldest in each group. In the dup(15) group, a 39-year-old female with autistic features and intractable epilepsy (onset at 9 years of age) and whose death was epilepsy-related had clusters of plaques in several neocortical regions, including the frontal, temporal and insular cortex (Fig. 7). Plaques were also found in the brains of two individuals diagnosed with idiopathic autism, including a 51-year-old subject who had had only one grand mal seizure (Fig. 8), and a 52-year-old individual whose records do not contain information about epilepsy or brain trauma. In both brains, the postmortem examination revealed numerous plaques within the entire cortical ribbon (Fig. S2) and in the amygdala, thalamus and subiculum (not shown).

In all three cases, thioflavin S staining did not reveal fluorescence in the plaques (not shown), suggesting that the amyloid plaques detected in the examined subjects with autism/dup(15) and idiopathic autism were nonfibrillar. However, positive immunoreactivity with all six antibodies used, including 6E10, 6F3, 4G8, Rabm38, Rabm40 and Rabm42 (Fig. 7 and 8) and 6F3D (not shown), revealed full-length Aβ_1–40/42_ peptides. In the plaque area, numerous glial cells, mainly with the morphology of astrocytes, and less numerous, glial cells with the morphology of microglial cells, contained Aβ-immunoreactive granular material. In contrast to the presence of full-length Aβ peptides in plaques, the Aβ peptides in both astrocytes and microglial cells in the plaque perimeter and surrounding tissue were mAb 6E10- and 6F3D-negative, indicating that they were the product of α-secretase. They were positive for the three other antibodies, Rabm38, Rabm40 and Rabm42, demonstrating that both astrocytes and microglia accumulate Aβ_17–40/42_.

**Figure 9:**
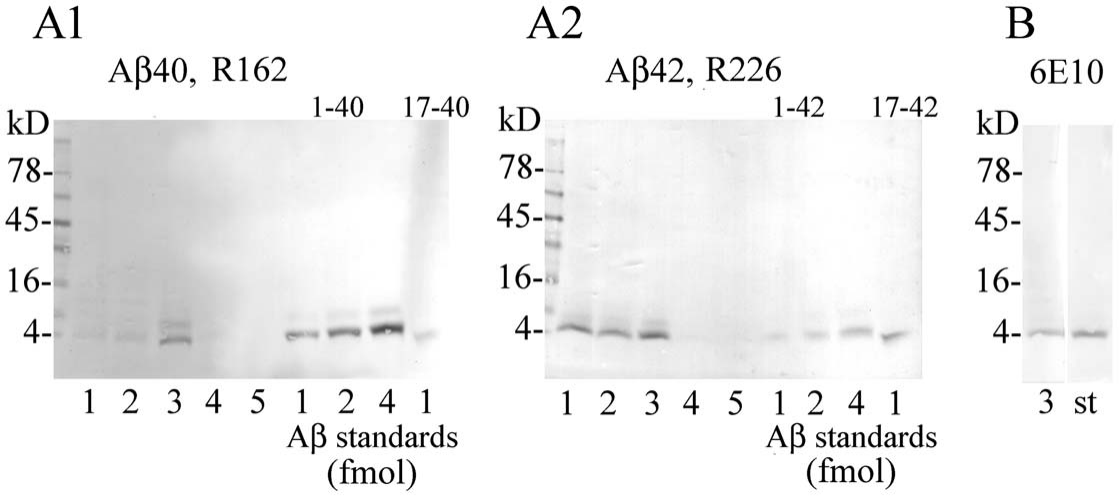
Properties of Aβ in plaque-rich cortex characterized by Western blotting. Panels A1 and A2 show Aβ40 and Aβ42 detected with pAbs R162 and R226, respectively, in blots of extracts (3 µg of total proteins per line) from cerebral cortex containing diffuse plaques of a 39-year-old subject with dup(15) (lane 1), of 51- and 52-year-old individuals with idiopathic autism (lanes 2 and 3), and of 48- and 47-year-old controls (lane 4 and 5). Blots reveal full-length Aβ, mainly Aβ42, in samples from plaque-positive subjects but not in controls. As standards, 1, 2 and 4 fmols of synthetic Aβ1–40, 17–40 (panel A1) and Aβ1–42, 17–42 (panel A2) were used. Panel B shows Aβ detected with mAb 6E10 specific for the N-terminal portion of Aβ in extract from the cortex of the 52-year-old subject (lane 3; 6 µg of protein per lane) and 4 fmol of synthetic Aβ1–40 (st). Panels A1, A2 and B demonstrate that in the extracts from diffuse plaque–positive cortical samples of autistic subjects, the levels of Aβ1–40 and 1–42 exceeded 1.5 fmol per 1 µg of extracted protein.

The extracts from the areas of the cerebral cortex in which diffuse plaques were detected by immunohistochemistry contained Aβ, mainly Aβ1–42, revealed by immunoblotting as a 4-kD band reacting with pAb R226 and mAb 6E10. The levels of Aβ1–42 in the samples exceeded 1.5 fmol per 1 µg of extracted proteins, whereas the levels of extracted Aβ 1–40 were low, below 0.2 fmol per 1 µg of extracted proteins (Fig. 9).

**Figure 10:**
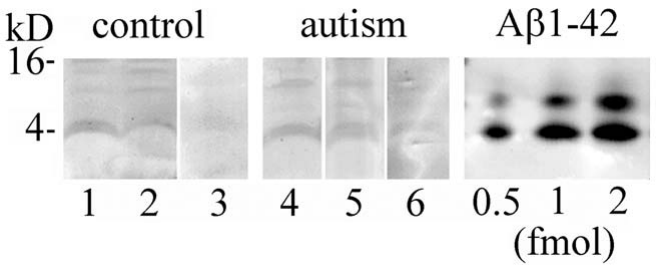
Detection by Western blots of Aβ in plaque-free subjects. Aβ42 detected with pAb R266 in lysates from cerebral cortex of control individuals 31, 32 and 51 years old (lanes 1–3, respectively), and individuals with idiopathic autism 8, 22, 29, and 29 years old (lane 4, 6, respectively). 40 µg of total lysate proteins were loaded per lane. Synthetic Aβ1–42 was used as a standard.

Immunoblotting of lysates from the cerebral cortex of autistic subjects without plaques and age-matched control subjects detected Aβ42 (Fig. 10) and Aβ40 (not shown) as a 3- to 4-kD band reacting with the pAb R226 and pAb R162, respectively. The levels of Aβ42 in the samples were in the range below 0.5 fmol per 40 µg of total proteins.

### Neurofibrillary Degeneration

A very few neurofibrillary tangles (NFTs) were found in the entorhinal cortex and amygdala in a 43-year-old control subject and in the entorhinal cortex and cornu Ammonis of a 47-year-old control subject. A few NFTs were found in the entorhinal cortex, CA1 and parasubiculum in a 51-year-old autistic subject and in the entorhinal and temporal cortex and the amygdala of a 52-year-old autistic subject. Neurofibrillary changes were not found in the dup(15) autism cohort with the oldest examined subject who died at the age of 39 years.

## Discussion

The accumulation of intraneuronal Aβ is considered a first step leading to amyloid plaque formation in AD [14], [22]–[24]. However, our examination of control brains during the life span showed that intraneuronal Aβ also occurs in normal controls and that almost all cytoplasmic Aβ peptides are the product of α- and γ-secretases (Aβ_17–40/42_) [21], whereas, the majority of amyloid in plaques is the product of β- and γ-secretases. This finding suggests that brain region– and neuron type–specific patterns of intraneuronal Aβ_17–40/42_ peptide accumulation in control brains are a baseline for detection and evaluation of increases associated with autism, FXS, epilepsy, brain trauma or age-associated neurodegeneration, such as AD.

### Detection of Aβ in Human Postmortem Material

The epitopes of mAbs 6E10 (4–13 aa of the Aβ sequence) and 4G8 (17–24 aa) are present in full-length APP and various APP fragments. Recently, Winton et al. [25] demonstrated that neuronal APP is immunolabelled with these two antibodies in mouse brain fixed for 24 hours in 10% neutral buffered formalin. However, the pattern of immunostaining in human brain fixed in formalin for at least several months, dehydrated almost 3 weeks in ascending concentrations of ETOH, and embedded in PEG indicates that mAbs 4G8, 6E10 and 6F3D do not detect APP in tissue subjected to this process. The role of technical factors in the loss of access of these antibodies to their epitopes in APP was previously documented in studies of tissue fixed in formalin for 10 days and in studies of cultured cells [26], [27]. Several observations in this report indicate that these antibodies do not detect APP. Massive immunolabelling of neuronal APP with R57 is in striking contrast with the presence of only traces of 6E10 and 6F3D immunoreactivity in these cells and the only partial co-localization of Aβ and APP labeling in the amyloid-rich neurons of autistic subjects. These data indicate that in the examined material, APP is detected with the APP-specific antibody R57, but not with mAbs 4G8, 6E10,and 6F3D, which, however, detect Aβ. One may assume that the epitopes of these antibodies, but not the R57 epitopes, are blocked or modified in APP molecules during long exposure to chemicals used for fixation, dehydration and embedding. Consistent with immunocytochemistry, Western blotting identifies 3–4 kD Aβ not only in subjects with diffuse plaques, but also in autistic subjects without plaques and in control subjects.

### Excessive Accumulation of Aβ_17–24_ in Neurons in Idiopathic Autism and dup(15) Autism

This is the first report documenting excessive accumulation of Aβ in the neurons of subjects with idiopathic autism and an even more pronounced accumulation in the dup(15) autism cohort. Two patterns of excessive accumulation distinguish these two cohorts from control subjects and indicate that excessive accumulation is neuron type/brain region–specific. Type 1 of altered Aβ accumulation is reflected in an increase in the percentage of neurons with strong Aβ accumulation by 7.6-fold in the amygdala and thalamus and by 4.5-fold in the LGB in individuals with dup(15) autism in comparison to the control group. A similar (by 5.3×, 6.3× and 3.9×, respectively) and statistically significant increase was found in the idiopathic autism group. Type 2 of altered Aβ accumulation is reflected in a more uniform increase in the percentage of neurons with combined strong, moderate and weak immunoreactivity. Again, this pattern is observed in both autistic cohorts in the pyramidal neurons in all three examined cortical regions.

These findings suggest that metabolic alterations are similar in both types of autism and that the severity of these alterations is less pronounced in idiopathic autism than in autism caused by dup(15). The significant increase in the percentage of neurons with enhanced cytoplasmic Aβ load in idiopathic autism and the fact that almost all of this Aβ is the product of α-secretase show the striking similarity to increased levels of sAPP-α in blood plasma in 60% of autistic children (6,16). In studies by Sokol et al. [6] and Ray et al. [16], aggressive behavior was identified as associated with increased levels of sAPP-α. Bailey et al. [9] also detected a significant increase in sAPP-α levels in 60% of autistic children but with no association between the severity of aggression, social or communication sub-scores and increased levels of sAPP-α. Due to the neurotrophic properties of sAPP-α, the authors proposed that an increased level of the products of α-secretase may help identify a subset of children in which early regional brain overgrowth is necessary and sufficient for the development of autism and may even represent a mechanism regulating overgrowth in autism. However, the most pronounced accumulation of amino-terminally truncated Aβ observed in the dup(15) autism cohort with microcephaly [28] indicates that intraneuronal Aβ accumulation of the products of α-secretase is not associated with brain overgrowth. Our data identify a dup(15) autism subcohort with microcephaly, more severe clinical phenotype, very early onset of seizures, a high percentage of intractable seizures, and a high prevalence of sudden unexpected death in epilepsy (SUDEP) as associated with the highest percentage of neurons accumulating α-secretase product.

### Trafficking of Aβ_17–24_ in Neurons

Aβ is generated in the endolysosomal pathway and in the endoplasmic reticulum/Golgi compartment [29]–[33] and is also detected in multivesicular bodies [34] and in mitochondria [15], [35]. The application of Lamp1 as a lysosomal marker revealed that approximately 20–30% of neuron cytoplasmic Aβ_17–24_ accumulates in this step of the proteolytic pathway in control and autistic subjects. An increase in cathepsin D protein expression, as reported in several brain regions of autistic subjects, suggests the selective enhancement of target proteins’ hydrolysis by this aspartic acid protease [36]. The lysosome is the major acid hydroxylase-containing cell compartment engaged in processing of substrates delivered by (a) endocytosis, (b) autophagy [37] and (c) scavenging of proteins from the endoplasmic reticulum to lysosomes [38]. The increase of Aβ_17–24_ in the lysosomes of autistic subjects may reflect Aβ_17–40/42_ generation in these pathways.

This study revealed that another 20–30% of neuron Aβ_17–40/42_ is present in lipofuscin, which is the final product of cytoplasmic proteolytic degradation of exogenous and endogenous substrates. During the entire lifespan, lipofuscin gradually accumulates in neurons [39]. The age of onset and dynamics of lipofuscin deposition are cell type–specific [40], [41]. Our previous study revealed that neurons in the inferior olive, dentate nucleus and lateral geniculate body start accumulating lipofuscin and Aβ_17–40/42_ early in life and that this accumulation progresses with age at region-specific rates [21]. The confocal microscopy study indicates that in spite of the known nonspecific binding of some antibodies to lipofuscin, the selection of the immunostaining protocol and the setting of proper thresholds in confocal imaging applied in this study reveal the selectivity of mAbs 4G8 and 6E10, and pAb R226 binding to some lipofuscin deposits.

The pattern of both Aβ and lipofuscin accumulation can be modified in early childhood in subjects with autism and even more significantly in individuals with dup(15) autism. The difference is detectable as an increase in the percentage of Aβ_17–40/42_ immunoreactive neurons, the amount of immunopositive material per neuron, and the number of brain regions and neuron types affected in both children and adults. Detected changes in Aβ accumulation may reflect abnormal accumulation of lipofuscin, as reported by Lopez-Hurtado and Prieto [42]. An increase in the number of lipofuscin-containing neurons by 69% in Brodmann area (BA) 22, by 149% in BA 39, and by 45% in BA 44, in brain tissue samples from autistic individuals 7 to 14 years of age, was observed together with a loss of neurons and glial proliferation. However, enhanced lipofuscin accumulation is not unique for idiopathic autism or autism/dup(15). It has been reported in Rett syndrome [43], an ASD, as well as in several psychiatric disorders, including bipolar affective disorder [44] and schizophrenia [45], [46].

Enhanced lipofuscin accumulation and enhanced Aβ_17–40/42_ immunoreactivity in the majority of the examined brain structures in most of the individuals with autism and the subjects with dup(15) may be a reflection of enhanced oxidative stress. Oxidative stress contributes to protein and lipid damage in cytoplasmic components, their degradation in lysosomal and autosomal pathways, and the deposition of products of degradation in lipofuscin or their exocytosis [47], [48]. The link between oxidative stress, cytoplasmic degradation and lipofuscin deposition is supported by the presence of oxidatively modified proteins and lipids in lipofuscin [39], [49], [50]. A significant increase in malondialdehyde levels (a marker of lipid peroxidation) in the plasma of autistic children [51] and in the cerebral cortex and cerebellum [52] may reflect oxidative damage leading to enhanced degradation, and the possible increased turnover of affected cell components.

### Biological Activity of N-terminally Truncated Aβ

The results of confocal microscopy suggest that on average, 30% of neuronal Aβ is present in lysosomes and another 30% in lipofuscin. However, the biological consequences of accumulation of Aβ, in the lysosomes or in lipofuscin are not known. N-terminally truncated Aβ peptides exhibit enhanced peptide aggregation relative to the full-length species [53] and retain their neurotoxicity and β-sheet structure. Soluble intracellular oligomeric Aβ (oAβ) species inhibit fast axonal transport (FAT) in both anterograde and retrograde directions [54]. Inhibition of FAT results from activation of endogenous casein kinase 2. Altered regulation of FAT markedly reduces transport of synaptic proteins and mitochondria in the AD brain and in AD mouse models that accumulate oAβ [55]. Dysregulation of FAT results in distal axonopathies with a reduced delivery of critical synaptic elements required for the integrity, maintenance and function of synapses [54].

The *in vitro* studies suggest that Aβ 17–24 is toxic to neurons. Treatment of SH-SY5Y and IMR-32 human neuroblastoma cells with Aβ 17–24 causes apoptotic death similar to in cells incubated with Aβ1–42, whereas treatment with Aβ17–40 results in a lower level of apoptosis, comparable to experimental exposure to Aβ1–40. This apoptosis is mediated predominantly by the caspase-8 and caspase-3 pathways [56]. However, *in vitro* studies of the neuronal response to exogenous Aβ peptides do not replicate the neuronal exposure to endogenous Aβ_17–40/42_ trafficking inside vesicles and vacuoles of lysosomal pathway.

### Aβ_1–40/42_ in Diffuse Plaques of Autistic Subjects

The presence of diffuse nonfibrillar plaques in two autistic subjects who were more than 50 years old and in one 39-year-old subject with autism/dup(15) suggests that in the fourth/fifth decade of life, there is an increased risk of the second type of changes: activation of the amyloidogenic pathway of APP processing with β- and γ-secretases, resulting in focal deposition of Aβ_1–40/42_ in plaques_._ It was hypothesized that Aβ_17–42_ peptides may initiate and/or accelerate plaque formation, perhaps by acting as nucleation centers that seed the subsequent deposition of relatively less amyloidogenic but apparently more abundant full-length Aβ [53], [57], [58]. Gouras et al. [59] considered intracellular Aβ_42_ accumulation an early event leading to neuronal dysfunction. The Aβ_1–40/42_ –positive diffuse plaques in the brains of autistic subjects are different from the Aβ_17–40/42_–positive cerebellar diffuse plaques detected in DS [57], [60]. Diffuse amorphous nonfibrillar Aβ deposits, called amorphous plaques [61], pre-plaques [62] or pre-amyloid deposits [63], are considered to be of neuronal origin [64]–[67] and are formed selectively in projection areas of distant affected neuronal populations [68]. Diffuse plaque formation in autistic subjects suggests the activation of the secretory pathway and the synaptic release of Aβ_1–40/42_.

The presence of Aβ_17–40/42_ in astrocytes in Aβ_1–40/42 –_positive diffuse plaques suggests that the full-length Aβ released by neurons is phagocytosed and processed by local astrocytes. One may hypothesize that the proliferation of Aβ-positive astrocytes, the increase of cytoplasmic Aβ immunorectivity in astrocytes, the presence of Aβ in all astrocytes in the affected region, astrocyte death and the deposition of large aggregates of extracellular Aβ in the cerebral cortex or hippocampus of autistic children and young adults is a response to the elevated levels of extracellular Aβ_17–40/42_ and/or Aβ_1–40/42_. Therefore, the number of Aβ-positive astrocytes may be an indicator of the local concentration of extracellular Aβ not only in plaque-positive but also in plaque-negative brain regions, occurring decades before plaque formation. Cytoplasmic granular immunoreactivity (Aβ17–23 and Aβ8–17) was reported in astrocytes in AD [69]. In astrocytes, intracellular Aβ appears in lysosomes and lipofuscin [70], [71]. It defines the role of astrocytes in the uptake of different species of Aβ in diffuse and neuritic plaques and their subsequent degradation in lysosomes and storage of products of degradation in lipofuscin [69].

In the examined autistic cohort, the early onset of intractable epilepsy and the epilepsy-related chronic and acute brain trauma appear to be additional risk factors for APP pathway activation and diffuse plaques formation. Repetitive brain trauma, including that related to epilepsy and head banging, produces a chronic traumatic encephalopathy with the associated deposition of Aβ, most commonly as diffuse plaques [72]–[74]. In acute traumatic brain injury, diffuse cortical Aβ deposits were detected in 30% to 38% of cases 2 hours after injury [75]–[77].

The presence of a few NFTs in the entorhinal cortex, cornu Ammonis and amygdala in 43- and 47-year-old control subjects and in these structures and in the parasubiculum and temporal cortex of 51- and 52-year-old autistic subjects is consistent with the topography and amount of age-associated neurofibrillary degeneration and NFT distribution observed in the general population [78].

In conclusion, this postmortem study of Aβ distribution in the brain of subjects with idiopathic autism and dup(15) autism suggests (a) very significant enhancement of intraneuronal Aβ accumulation in almost all examined cortical and subcortical structures in autism, especially in autism associated with dup(15); (b) the prevalence of anabolic α-secretase APP processing and Aβ_17–40/42_ accumulation in neuronal endosomes, autophagic vacuoles, lysosomes and lipofuscin in the majority of autistic children and adults; and (c) activation of the amyloidogenic pathway of APP processing with β- and γ-secretases in the late adulthood of some autistic subjects with diffuse nonfibrillar plaque formation and astrocyte activation.

## Materials and Methods

### Material, Clinical and Genetic Evaluation

The brains studied were from nine individuals diagnosed with dup(15) ages 9 to 39 years (five males and four females), 11 subjects with idiopathic autism ages 2 to 52 years (10 males and one female), and eight control subjects ages 8 to 47 years (four males and four males) (Table 1). Medical records were obtained following consent for release of information from the subjects’ legal guardians. The study was approved by the Institutional Review Boards for the New York State Institute for Basic Research in Developmental Disabilities; the University of California, Los Angeles; and Nemours Biomedical Research, duPont Hospital for Children, Wilmington. Clinical and genetic studies were performed as described previously [28]. Clinical characteristics were based on psychological, behavioral, neurological and psychiatric evaluation reports. To confirm a clinical diagnosis of autism, the Autism Diagnostic Interview-Revised (ADI-R) was administered to the donor family [79].

**Table 1:** Material examined, cause of death, and the prevalence of epilepsy.

Group	Brain Bank number	Sex	Age (y)	Cause of death	Epilepsy. age of onset
dup(15)	AN14762	M	9	SUDEP	IE/10 m
dup(15)	AN06365	M	10	SUDEP	IE/8 m
dup(15)	AN09402	M	11	SUDEP	IE/10 m
dup(15)	AN07740	F	15	SUDEP	E/11 y
dup(15)	AN09470	F	15	Aspiration pneumonitis	–
dup(15)	AN03935	M	20	Cardiopulmonary arrest	–
dup(15)	AN05983	M	24	Pneumonia	IE/7 y
dup(15)	AN14829	F	26	SUDEP	E/16 y
dup(15)	AN11931	F	39	SUDEP	IE/9 y
Autism	AN03345	M	2	Asphyxia (drowning)	–
Autism	AN13872	F	5	Asphyxia (drowning)	–
Autism	AN08873	M	5	Asphyxia (drowning)	–
Autism	HSB4640	M	8	Asthma attack	E/8 y
Autism	AN01293	M	9	Heart failure	–
Autism	CAL105	M	11	Asphyxia (drowning)	E
Autism	IBR93-01	M	23	Seizure related	E/23 y
Autism	AN08166	M	28	Seizure-related	E
Autism	NP06-54	M	32	Brain tumor	–
Autism	AN17254	M	51	Heart failure	1 grand mal
Autism	BB1376	M	52	Heart failure	–
Control	UMB1706	F	8	Rejection of cardiac transplant	–
Control	UMB1670	M	14	Asphyxia (hanging)	–
Control	UMB4722	M	14	Multiple traumatic injuries	–
Control	BTB3960	F	25	Not known	–
Control	IBR291-00	M	32	Heart failure	–
Control	IBR212-98	F	33	Bronchopneumonia	–
Control	IBR38-98	F	43	Sepsis	–
Control	IBR457-96	M	47	Myocardial infarct	–

Sudden unexpected and unexplained death of subject with known epilepsy (SUDEP), Intractable epilepsy (IE), Epilepsy (E), Years (y), Months (m).

Molecular genetic evaluations, using antemortem peripheral blood samples and lymphoblast cell lines for eight of the dup(15) cases, included genotyping with 19–33 short tandem repeat polymorphisms from chromosome 15, Southern blot analysis of dosage with 5–12 probes, measurement of the methylation state at *SNRPN exon α*, as described [80], and array comparative genomic hybridization [81]. Duplication morphology was confirmed by fluorescent in situ hybridization [80].

In eight cases, tetrasomy, and in one case, hexasomy of the Prader-Willi/Angelman syndrome critical regions was detected. In eight cases, the origin of abnormality was maternal; in one case, the origin was not determined. In the examined dup(15) group, seven of nine subjects (78%) were diagnosed with autism or ASD, and seven had seizures. In six cases (67%), SUDEP was reported. In the idiopathic autism cohort, two subjects (8-year-old male, HSB4640, and 52-year-old male, BB1376), were diagnosed with the ASD (pervasive developmental disorder – not otherwise specified and high-functioning atypical autism, respectively). In all other cases, the clinical diagnosis of autism was confirmed with ADI-R.

One brain hemisphere was preserved for neuropathological and immunocytochemical studies. Methods and results of neuropathological evaluations of developmental abnormalities have been summarized in our previous reports [28], [82]. The mean postmortem interval varied from 23.9 h in the dup(15) cohort to 19.6 h in the idiopathic autism cohort and 15.0 h in the control group. One brain hemisphere from each subject was fixed in 10% buffered formalin for a period ranging from six weeks to several months, dehydrated in a graded series of ethanol, infiltrated and embedded with PEG (Sigma) [83] and stored at 4°C. Tissue blocks were then cut into 50-µm-thick serial sections and stored in 70% ethyl alcohol. Two brains (AN17254 and BB1376) were embedded in celloidin, as described [82] and were cut alternatively into 200- and 50-µm-thick serial sections.

### Immunocytochemistry and Confocal Microscopy

Brain Bank identification of the tissue samples is listed in Table 1, to maintain non-overlapping records of results of brains examined in different projects. Immunocytochemistry and confocal microscopy were applied to characterize (a) Aβ distribution in cells in the cerebral cortex, subcortical structures, cerebellum and brainstem and in diffuse plaques; (b) the Aβ peptide properties; and (c) Aβ distribution in endosomes, lysosomes, autophagic vacuoles, mitochondria and lipofuscin (Table 2).

**Table 2:** Antibodies used for immunocytochemistry, immunofluorescence and western blotting.

Name	Epitope or target	Dilution	Host	Application	Source
6E10	4–13 aa Aβ	1∶10,000	M-m	ICH,CM, WB	Covance, Inc., Princeton, Inc. [84], [85]
6F3D	8–17 aa Aβ	1∶50	M-m	ICH	Novocastra Laboratories Ltd., Newcastle, UK
4G8	17–24 aa Aβ	1∶8,000	M-m	ICH,CM, WB	IBR [86]
Rabm38	−38 aa Aβ	100 ng/mL	R-m	ICH	IBR
Rabm40	−40 aa Aβ	100 ng/mL	R-m	ICH	IBR [87]
Rabm42	−42 aa Aβ	100 ng/mL	R-m	ICH	IBR [87]
R57	APP C-terminal aa 671–695	1∶3,000	R-p	CM	IBR
R226	36–42 aa Aβ		R-p	CM, WB	IBR
R162	Aβ C-terminus		R-p	CM, WB	IBR
LAMP 1	Lysosomes	1∶400	R-p	CM	Abgent, San Diego, CA
Rab5Ab13253	Early endosomes	1∶100	R-p	CM	Abcam Inc., Cambridge, MA
LC3B	Autophagic vacuoles	1∶100	R-m	CM	Cell Signaling Technology Inc., Danvers, MA
COXIV	Mitochondria	1∶100	R-m	CM	Cell Signaling Technology Inc., Danvers, MA
GFAP	Astrocytes	1∶400	R-p	CM	Sigma, Saint Louis, MO
AIF/IBA1AF1039C	Microglia	1∶200	G-p	CM	Abgent, San Diego, CA
Tau-1	Tau protein	1∶1000	M-m	ICH	IBR

Mouse monoclonal (M-m), Rabbit monoclonal (R-m) or polyclonal (R-p), Goat polyclonal (G-p). Immunocytochemistry (ICH), Confocal microscopy (CM), Western blots (WB).

mAbs 6E10 (Covance, Inc., Princeton, Inc.) and 6F3D (Novocastra Lab. Ltd., Newcastle, UK) were used to characterize the N-terminal portion of Aβ. mAb 6E10 recognizes an epitope in residues 4–13 of Aβ [84], [85]. mAb 6F/3D recognizes an epitope in residues 8–17 of Aβ. The middle portion of Aβ was detected with mAb 4G8, which recognizes an epitope in residues 17–24 of Aβ [86]. The carboxyl terminus of Aβ was characterized with rabbit monoclonal antibodies Rabm38, Rabm40 and Rabm42, which detect Aβ_−38_, Aβ_−40_, and Aβ_−42_, respectively [87]. The specificity of mAbs 4G8 and 6E10 for Aβ was verified in the examined postmortem human brain tissue by double immunolabeling with pAb R57 detecting APP C-terminal aa 671–695.

To detect intracellular Aβ peptides and amyloid in plaques, free-floating sections were treated with 70% formic acid for 20 minutes [88]. The endogenous peroxidases in the sections were blocked with 0.2% hydrogen peroxide in methanol. The sections were then treated with 10% fetal bovine serum in phosphate buffer solution (PBS) for 30 minutes to block nonspecific binding. The primary antibodies were diluted in 10% fetal bovine serum in PBS and sections were treated overnight at 4°C. The sections were washed and treated for 30 min with either biotinylated sheep anti-mouse IgG antibody or biotinylated donkey anti-rabbit IgG antibody diluted 1∶200. The sections were treated with an extravidin peroxidase conjugate (1∶200) for 1 h, and the product of reaction was visualized with diaminobenzidine (0.5 mg/mL with 1.5% hydrogen peroxide in PBS). After immunostaining, sections were lightly counterstained with cresyl violet. To detect fibrillar Aβ in plaques, sections were stained with Thioflavin S and examined in fluorescence.

Neurons with fibrillary tangles were immunolabelled with mAb Tau-1, detecting an epitope between amino acids 189 and 207 of the human tau protein sequence [89]. To detect abnormally phosphorylated tau with Tau-1, sections were pretreated with alkaline phosphatase (Sigma, Saint Louis, MO; Type VII-L, 400 µg/ml in PBS, pH 7.4, 0.01% H_2_0_2_).

Double immunofluorescence for Aβ (mAb4G8) and for astrocytes (GFAP; rabbit polyclonal antibody, pAb, Sigma) was carried out to confirm the presence of Aβ in astrocytes. Confocal microscopy was applied to detect Aβ localized in neuronal cytoplasmic organelles. Aβ in lysosomes was detected by using lysosomal-associated membrane protein marker (LAMP1; Abgent, San Diego, CA). Early endosomes were immunodetected with rabbit pAb Rab5 (Ab13253; Abcam, Cambridge, MA), whereas autophagic vacuoles were immunolabelled with rabbit mAb LC3B (Cell Signaling Technology Inc., Danvers, MA). Mitochondria were detected with the rabbit mAb COXIV Alexa Fluor 488 conjugated (Cell Signaling Technology). To detect Aβ, brain sections were treated with 70% formic acid for 20 min, washed in PBS 2x 10 min and double- immunostained using mAb 4G8 and antibodies detecting markers of cytoplasmic organelles. Affinity-purified donkey antisera against mouse IgG labeled with Alexa Fluor 488 and against rabbit IgG labeled with Alexa Fluor 555 (both from Molecular Probes/Invitrogen) were used as secondary antibodies. TO-PRO-3-iodide (Molecular Probes/Invitrogen) was used to counterstain cell nuclei. Absence of cross-reaction was confirmed as previously described [26]. Images were generated using a Nikon C1 confocal microscope system with EZC1 image analysis software.

### Comparison of Intraneuronal Aβ Accumulation in Examined Cohorts

Semiquantitative estimation of intraneuronal Aβ was performed without knowledge of the subject’s age, gender or clinical diagnosis or the neuropathological diagnosis of the tissue being analyzed. Evaluation was performed at a workstation consisting of Axiophot II light microscope, specimen stage with 3-axis computer-controlled stepping motor system (Ludl Electronics; Hawthorne, NY), CCD color video camera (CX9000 MicroBrightField Bioscience, Inc., Williston, VT) and stereology software (Stereo Investigator, MicroBrightField Bioscience Inc.). Grid size and the virtual test area were designated individually for each brain region to adjust to the region of interest size and shape. Intraneuronal Aβ accumulation has been estimated by four neuropathologists in 12 brain structures including frontal, temporal and occipital cortex, amygdala, thalamus, lateral geniculate body, sectors CA1 and CA4, and dentate gyrus in the hippocampal complex, Purkinje cells and dentate nucleus in cerebellum, and inferior olive in the brainstem. The number of 4G8-negative neurons and neurons with weak (<10 immunopositive granules per cell), strong (condensed mass of indistinguishable small and large immunoreactive granules) and medium (>weak and <strong) immunoreactivity was determined using a ×40 objective lens. For each subject, from 100 to 180 neurons were examined per region of interest in sections immunostained with mAb 4G8. Inspection of the entire cell cytoplasm by using micrometer screw contributed to precise rating of amyloid load in each examined neuron.

Differences in the estimated cytoplasmic neuronal Aβ load were examined using the Mann-Whitney U (Wilcoxon signed ranks) test or, for comparison of all three groups, the Kruskal-Wallis one-way ANOVA (an extension of the U test) [90]. Statistics were computed from pooled data from each group [dup(15) autism, idiopathic autism, control], where sampled neurons immunoreactivity was categorized as strong, medium, weak or none.

### Western Blotting

Frozen temporal cortex samples from three control and three autistic subjects were homogenized in 10×volume of 10 mM TRIS buffer containing 0.65% NP-40, 1 mM EDTA and Complete protease inhibitor coctail (Roche, Mannheim, Germany) in a Potter-Elvehjem homogenizer and sonicated for 2 minutes. Protein content in lysates was measured by BCA assay (Pierce). Forty µg of total lysate proteins were loaded per lane for PAGE in 8–15% gradient gels.

Tissue samples from formalin-fixed PEG or celloidin-embedded brains of three subjects with diffuse plaques detected by immunocytochemistry (39-year-old female diagnosed with dup(15) autism, a 51-year-old autistic subject, and a 52-year-old subject with atypical autism) and two subjects without plaques (48-year-old autistic and a 47-year-old control subject) were used for protein extraction. From 50-µm-thick sections, approximately 120 mm^2^ of affected cortex was dissected (approximately 6 mm^3^ of tissue), rehydrated in PBS and homogenized in Potter-Elvehjem homogenizer in PBS containing 0.5% sodium deoxycholate, 0.1% SDS and 1% NP-40 (RIPA buffer). After sonication two times for three minutes, the material was centrifuged at 16,000g for 20 minutes, and supernatants were collected as RIPA extracts. Protein content in the extracts was measured by the BCA assay (Thermo Scientific, Rockford, IL). For Aß detection with R162, R226, and mAb 6E10, the amounts of extracted proteins loaded per lane were 3, 3 and 6 µg, respectively. The proteins were subjected to PAGE in 8–15% gradient gels, transferred onto nitrocellulose and probed with antibodies specific for C-terminus of Aß40 (R162) and Aß42 (R226), and N-terminus–specific mAb 6E10.

## Supporting Information

Figure S1 **Neurons with low and high amyloid load.** In control brains, the percentage of Aβ-positive neurons and their amyloid load is much lower in CA1 than in CA4 sector and is very low in the granule neurons in the dentate gyrus. The percentage of Aβ-positive neurons and amyloid load is significantly higher in the dup(15) autism cohort than in the control and idiopathic autism groups (p<0.0001), but the difference between idiopathic autism and control is insignificant. The characteristic feature of the LGB, inferior olive and dentate nucleus of control subjects is the very high percentage of Aβ-positive neurons and the highest amyloid load among the examined 12 structures. The increase of amyloid load is undetectable in the inferior olive and is minimal in the LGB and dentate nucleus of subjects with idiopathic autism and dup(15) autism.
(TIF)

Figure S2 **Topography and morphology of neocortical diffuse plaques.** Low magnification demonstrates diffuse plaques immunostained with mAb4G8 (17–24 aa) in frontal, temporal and occipital cortex (FC, TC and OC, respectively) in the brain of a 39-year-old female diagnosed with dup(15) autism, a 51-year-old autistic male, and a 52-year-old subject with atypical autism.
(TIF)

## References

[1] American Psychiatric, Statistical Manual of Mental Disorders DSM-IV-TR (2000). Washington, DC: American Psychiatric Association.

[2] Rineer S, Finucane B, Simon EW (1998). Autistic symptoms among children and young adults with isodicentric chromosome 15.. Am J Med Genet.

[3] Simon EW, Finucane B, Rineer S (2000). Autistic symptoms in isodicentric 15 syndrome: response to Wolpert, et al.. Am J Med Genet (Neuropsychiat Genet).

[4] Hagerman RJ, Hagerman RJ, Hagerman PJ (2002). The physical and behavioral phenotype.. Fragile X syndrome: diagnosis, treatment, and research.

[5] Kent L, Evans J, Paul M, Sharp M (1999). Comorbidity of autistic spectrum disorders in children with Down syndrome.. Dev Med Child Neurol.

[6] Sokol DK, Chen D, Farlow MR, Dunn DW, Maloney B (2006). High levels of Alzheimer beta- amyloid precursor protein (APP) in children with severely autistic behavior and aggression.. J Child Neurol.

[7] Westmark CJ, Malter JS (2007). FMRP mediates mGluR5-dependent translation of amyloid precursor protein.. PLoS One Biology.

[8] Westmark CJ, Westmark PR, O’Riordan KJ, Ray BC, Hervey CM (2011). Reversal of fragile X phenotypes by manipulation of AβPP/Aβ levels in *Fmr1KO* mice.. PloS One.

[9] Bailey AR, Giunta BN, Obregon D, Nikolic WV, Tiaqn J (2008). Peripheral biomarkers in autism: secreted amyloid precursor protein-α as a probable key player in early diagnosis.. Int J Clin Exp Med.

[10] Sokol DK, Maloney B, Long JM, Ray B, Lahiri DK (2011). Autism, Alzheimer disease, and fragile X. APP, FMRP, and mGluR5 are molecular links.. Neurology.

[11] Iversen LL, Mortishire-Smith RJ, Pollack SJ, Shearman MS (1995). The toxicity in vitro of beta-amyloid protein. (Review).. Biochem J.

[12] Selkoe DJ (2001). Alzheimer’s disease: genes, proteins, and therapy.. Physiol Rev.

[13] Sevalle J, Amoyel A, Robert P (2009). Aminopeptidase A contributes to the N-terminal truncation of amyloid beta-peptide.. J Neurochem.

[14] Gouras GK, Tampellini D, Takahashi RH, Capetillo-Zarate E (2010). Intraneuronal β-amyloid accumulation and synapse pathology in Alzheimer’s disease.. Acta Neuropathol.

[15] Bayer TA, Wirths O (2010). Intracellular accumulation of amyloid-beta–a predictor of synaptic dysfunction and neuron loss in Alzheimer’s disease.. Front Aging Neurosci.

[16] Ray B, Long JM, Sokol DK, Lahiri DK (2011). Increased secreted amyloid precursor protein-α (sAPPα) in severe autism: proposal of a specific, anabolic pathway and putative biomarker.. PloS One 6: e20405,.

[17] Westmark CJ, Westmark PR, Malter JS (2010). MPEP reduces seizure severity in Fmr-1 KO mice overexpressing human Aβ.. Int J Clin Exp Pathol.

[18] Tuchman RF, Rapin I (2002). Epilepsy in autism.. Lancet Neurol.

[19] Moechars D, Lorent K, De Strooper B, Dewachter I, Van Leuven F (1996). Expression in brain of amyloid precursor protein mutated in the alpha-secretase site causes disturbed behavior, neuronal degeneration and premature death in transgenic mice.. EMBO J.

[20] Westmark CJ, Westmark PR, Beard AM, Hildebrandt SM, Malter JS (2008). Seizure susceptibility and mortality in mice that over-express amyloid precursor protein.. Int J Clin Exp Pathol.

[21] Wegiel J, Kuchna I, Nowicki K, Frackowiak J, Mazur Kolecka B (2007). Intraneuronal Aβ immunoreactivity is not a predictor of brain amyloidosis-β or neurofibrillary degeneration.. Acta Neuropath.

[22] Mochizuki A, Tamaoka A, Shimohata A, Komatsuzaki Y, Shoji S (2000). Aβ42-positive non-pyramidal neurons around amyloid plaques in Alzheimer’s disease.. Lancet.

[23] Gyure KA, Durham R, Stewart WF, Smialek JE, Troncoso JC (2001). Intraneuronal Aβ-amyloid precedes development of amyloid plaques in Down syndrome.. Arch Pathol Lab Med.

[24] D’Andrea MR, Nagele RG, Wang H-Y, Peterson PA, Lee DHS (2001). Evidence that neurons accumulating amyloid can undergo lysis to form amyloid plaques in Alzheimer’s disease.. Histopathology.

[25] Winton MJ, Lee EB, Sun E, Wong MM, Leight S (2011). Intraneuronal APP, not free Aβ peptides in 3xTg-AD mice: implications for tau versus Aβ-mediated Alzheimer neurodegeneration.. J Neurosci.

[26] Frackowiak J, Miller DL, Potempska A, Sukontasup T, Mazur-Kolecka B (2003). Secretion and accumulation of Aβ by brain vascular smooth muscle cells from A βPP-Swedish transgenic mice.. J Neuropathol Exp Neurol.

[27] Frackowiak J, Sukontasup T, Potempska A, Mazur-Kolecka B (2004). Lysosomal deposition of Aβ in cultures of brain vascular smooth muscle cells is enhanced by iron.. Brain Res.

[28] Wegiel J, Schanen NC, Cook EH, Sigman M, Brown WT (2012). Difference between the patterns of developmental abnormalities in autism associated with duplications 15q11.2q13 and idiopathic autism..

[29] Cook DG, Forman MS, Sung JC, Leight S, Kolson DL (1997). Alzheimer’s Aβ (1–42) is generated in the endoplasmic reticulum/intermediate compartment of NT2N cells.. Nat Med.

[30] Hartmann T, Bieger SC, Bruhl B, Tienari PJ, Ida N (1997). Distinct sites of intracellular production for Alzheimer’s disease Aβ40/42 amyloid peptides.. Nat Med.

[31] Greenfield JP, Tsai J, Gouras GK, Hai B, Thinakaran G (1999). Endoplasmic reticulum and trans-Golgi network generate distinct populations of Alzheimer β-amyloid peptides.. Proc Natl Acad Sci U S A.

[32] Glabe C (2001). Intracellular mechanisms of amyloid accumulation and pathogenesis in Alzheimer’s disease.. J Mol Neurosc.

[33] Wilson CA, Doms RW, Lee VM-Y (1999). Intracellular APP processing and Aβ production in Alzheimer disease.. J Neuropathol Exp Neurol.

[34] Takahashi RH, Milner TA, Li F, Nam EN, Edgar MA (2002). Intraneuronal Alzheimer Aβ42 accumulates in multivesicular bodies and is associated with synaptic pathology.. Am J Pathol.

[35] Caspersen C, Wang N, Yao J, Sosunov A, Chen X (2005). Mitochondrial Aβ: a potential focal point for neuronal metabolic dysfunction in Alzheimer’s disease.. FASEB J.

[36] Sheikh AM, Li X, Wen G, Tauqeer Z, Brown WT (2010). Cathepsin D and apoptosis related proteins elevated in the brain of autistic subjects.. Neuroscience.

[37] Gordon PB, Hoyvik H, Seglen PO (1992). Prelysosomal and lysosomal connections between autophagy and endocytosis.. Biochem J.

[38] Noda T, Farquhar MG (1992). A non-autophagic pathway for diversion of ER secretory proteins to lysosomes.. J Cell Biol.

[39] Brunk UT, Terman A (2002). Lipofuscin: mechanisms of age-related accumulation and influence on cell function.. Free Radic Biol Med.

[40] Brody H (1960). The deposition of aging pigment in the human cerebral cortex.. J Geront.

[41] Bancher C, Grundke-Iqbal I, Kim KS, Wisniewski HM (1989). Immunoreactivity of neuronal lipofuscin, with monoclonal antibodies to the amyloid β-protein.. Neurobiol Aging.

[42] Lopez-Hurtado E, Prieto JJ (2008). A microscopic study of language-related cortex in autism.. Am J Biochem Biotechn.

[43] Jellinger K, Armstrong D, Zoghbi HY, Percy AK (1988). Neuropathology of Rett syndrome.. Acta Neuropathol.

[44] Yanik M, Vural H, Tutkun H, Zoroglu SS, Savas HA (2004). The role of the arginine-nitric oxide pathway in the pathogenesis of bipolar affective disorder.. Eur Arch Psychiatry Clin Neurosci.

[45] Herken H, Uz E, Ozyurt H, Sogut S, Virit O (2001). Evidence that the activities of erythrocyte free radical scavenging enzymes and the products of lipid peroxidation are increased in different forms of schizophrenia.. Mol Psychiatry.

[46] Akyol O, Herken H, Uz E, Fadillioglu E, Unal S (2002). The indices of endogenous oxidative and antioxidative processes in plasma from schizophrenic patients: the possible role of oxidant/antioxidant imbalance.. Prog Neuropsychopharmacol Biol Psychiatry.

[47] Sohal RS, Brunk UT (1989). Lipofuscin as an indicator of oxidative stress and aging.. Adv Exp Med Biol.

[48] Brunk U T, Jones CB, Sohal RS (1992). A novel hypothesis of lipofuscinogenesis and cellular aging based on interactions between oxidative stress and autophagocytosis.. Mutat Res.

[49] Brunk UT, Terman A (2002). The mitochondrial-lysosomal axis theory of aging: accumulation of damaged mitochondria as a result of imperfect autophagocytosis.. Eur J Biochem.

[50] Terman A, Brunk UT (2004). Lipofuscin.. Int J Biochem Cell Biol.

[51] Chauhan A, Chauhan V, Brown WT, Cohen I (2004). Oxidative stress in autism: increased lipid peroxidation and reduced serum levels of ceruloplasmin and transferrin–the antioxidant proteins.. Life Sci.

[52] Chauhan V, Chauhan A, Chauhan A, Chauhan V, Brown WT (2010). Abnormalities in membrane lipids, membrane-associated proteins, and signal transduction in autism.. Autism.

[53] Pike CJ, Overman MJ, Cotman CW (1995). Amino-terminal deletions enhance aggregation of β-amyloid peptides *in vitro*.. J Biol Chem.

[54] Pigino G, Morfini G, Atagi Y, Deshpande A, Yu C (2009). Disruption of fast axonal transport is a pathogenic mechanism for intraneuronal amyloid beta.. PNAS.

[55] Pigino G, Morfini G, Mattson MP, Brady ST, Busciglio J (2003). Alzheimer’s presenilin 1 mutations impair kinesin-based axonal transport.. J Neurosci.

[56] Wei W, Norton DD, Wang X, Kusiak JW (2002). Aβ 17–42 in Alzheimer’s disease activates JNK and caspase-8 leading to neuronal apoptosis.. Brain.

[57] Gowing E, Roher AE, Woods AS, Cotter RJ, Chaney M (1994). Chemical characterization of Aβ17–42 peptide, a component of diffuse amyloid deposits of Alzheimer disease.. J Biol Chem.

[58] Saido TC, Iwatsubo T, Mann DMA, Shimada H, Ihara Y (1995). Dominant and differential deposition of distinct β-amyloid peptide species, AβN3(pE), in senile plaques.. Neuron.

[59] Gouras GK, Tsai J, Naslund J, Vincent B, Edgar M (2000). Intraneuronal Aβ42 accumulation in human brain.. Am J Pathol.

[60] Lalowski M, Golabek A, Lemere CA, Selkoe DJ, Wisniewski HM (1996). The “nonamyloidogenic “ p3 fragment (amyloid β 17–24) is a major constituent of Down’s syndrome cerebellar preamyloid.. J Biol Chem.

[61] Rozemuller JM, Eikelenboom P, Stam FC, Beyreuther K, Masters CL (1989). A4 protein in Alzheimer’s disease: primary and secondary cellular events in extracellular amyloid deposition.. J Neuropathol Exp Neurol.

[62] Mann DMA, Brown AMT, Prinja D, Davies CA, Landon M (1989). An analysis of the morphology of senile plaques in Down’s syndrome patients of different ages using immunocytochemical and lectin histochemical techniques.. Neuropathol Appl Neurobiol.

[63] Tagliavini F, Giaccone G, Linoli G, Frangione B, Bugiani O (1989). Cerebral extracellular preamyloid deposits in Alzheimer’s disease, Down syndrome and nondemented elderly individuals.. Prog Clin Biol Res.

[64] Dickson DW (1997). The pathogenesis of senile plaques.. J Neuropath Exp Neurol.

[65] Probst A, Langui D, Ipsen S, Robakis N, Ulrich J (1991). Deposition of beta/A4 protein along neuronal plasma membranes in diffuse senile plaques.. Acta Neuropathol.

[66] Wisniewski HM, Wegiel J, Kotula L (1996). Some neuropathological aspects of Alzheimer disease and its relevance to other disciplines.. Neuropath Appl Neurob.

[67] Wisniewski HM, Sadowski M, Jakubowska-Sadowska K, Tarnawski M, Wegiel J (1998). Diffuse, lake-like amyloid- ß deposits in the parvopyramidal layer of the presubiculum in Alzheimer disease.. J Neuropat Exp Neurol.

[68] Wegiel J, Wisniewski H (1999). Projections of neurons in neuritic plaques formation.. NeuroScience News.

[69] Thal DR, Härtig W, Schober R (1999). Diffuse plaques in the molecular layer show intracellular Aβ8–17 immunoreactive deposits in subpial astrocytes.. Clin Neuropath.

[70] Funato H, Yoshimura M, Yamazaki T, Saido TC, Ito Y (1998). Astrocytes containing amyloid β-protein (Aβ)-positive granules are associated with Aβ40-positive diffuse plaques in the aged human brain.. Am J Pathol.

[71] Yamaguchi H, Sugihara S, Ogawa A, Saido TC, Ihara Y (1998). Diffuse plaques associated with astroglial amyloid β protein, possibly showing a disappearing stage of senile plaques.. Acta Neuropathol.

[72] DeKosky ST, Abrahamson EE, Ciallella JR (2007). Association of increased cortical soluble Aβ42 levels with diffuse plaques after severe brain injury in humans.. Arch Neurol.

[73] Gentleman SM, Greenberg BD, Savage MJ, Noori M, Newman SJ (1997). A beta 42 is the predominant form of amyloid beta-protein in the brains of short-term survivors of head injury.. Neuroreport.

[74] McKee AC, Cantu RC, Nowinski CJ, Hedley-Whyte T, Gavett BE (2009). Chronic traumatic encephalopathy in athletes: progressive tauopathy after repetitive head injury.. J Neuropathol Exp Neurol.

[75] Roberts GW, Gentleman SM, Lynch A, Murray L, Landon M (1994). Beta amyloid protein deposition in the brain after severe head injury: Implications for the pathogenesis of Alzheimer’s disease.. J Neurol Neurosurg Psychiatry.

[76] Murakami N, Yamaki T, Iwamoto Y, Sakakibara T, Kobori N (1998). Experimental brain injury induces expression of amyloid precursor protein, which may be related to neuronal loss in the hippocampus.. J Neurotrauma.

[77] Ikonomovic MD, Uryu K, Abrahamson EE (2004). Alzheimer’s pathology in human temporal cortex surgically excised after severe brain injury.. Exp Neurol.

[78] Ohm TG, Müller H, Braak H, Bohl J (1995). Close-meshed prevalence rates of different stages as a tool to uncover the rate of Alzheimer’s disease-related neurofibrillary changes.. Neuroscience.

[79] Lord C, Rutter M, Le Couteur A (1994). Autism Diagnostic Interview-Revised: a revised version of a diagnostic interview for caregivers of individuals with possible pervasive developmental disorders.. J Autism Dev Disord.

[80] Mann SM, Wang NJ, Liu DH, Wang L, Schultz RA (2004). Supernumerary tricentric derivative chromosome 15 in two boys with intractable epilepsy: another mechanism for partial hexasomy.. Hum Genet.

[81] Wang NJ, Liu D, Parokonny AS, Schanen NC (2004). High-resolution molecular characterization of 15q11-q13 rearrangements by array comparative genomic hybridization (array CGH) with detection of gene dosage.. Am J Hum Genet.

[82] Wegiel J, Kuchna I, Nowicki K, Imaki H, Wegiel J (2010). The neuropathology of autism: defects of neurogenesis and neuronal migration, and dysplastic changes.. Acta Neuropath.

[83] Iqbal K, Braak H, Braak E, Grundke-Iqbal I (1993). Silver labeling of Alzheimer neurofibrillary changes and brain β-amyloid.. J Histotech.

[84] Kim KS, Wen GY, Bancher C, Chen CMJ, Sapienza VJ (1990). Detection and quantitation of amyloid β-peptide with 2 monoclonal antibodies.. Neurosci Res Comm.

[85] Miller DL, Currie JR, Mehta PD, Potempska A, Hwang Y-W (2003). Humoral immune response to fibrillar β-amyloid peptide.. Biochemistry.

[86] Kim KS, Miller DL, Sapienza VJ, Chen CMJ, Bai C (1988). Production and characterization of monoclonal antibodies reactive to synthetic cerebrovascular amyloid peptide.. Neurosci Res Commun.

[87] Miller DL, Potempska A, Wegiel J, Mehta PD (2011). High-affinity rabbit monoclonal antibodies specific for amyloid peptides amyloid-β40 and amyloid-β42 J Alz Dis.

[88] Kitamoto T, Ogomori K, Tateishi J, Prusiner S (1987). Methods in laboratory investigation. Formic acid pretreatment enhances immunostaining of cerebral and systemic amyloids.. Lab Invest.

[89] Goedert M, Spillantini M, Jakes R, Rutherford D, Crowther R (1989). Multiple isoforms of human microtubule-associated protein tau: sequences and localization in neurofibrillary tangles of Alzheimer’s disease.. Neuron.

[90] Siegal S, Castellan NJ (1988). Nonparametric statistics for the behavioral sciences, 2nd ed..

