# Supplementary figures and images for: Abnormal Intracellular Accumulation and Extracellular Aβ Deposition in Idiopathic and Dup15q11.2-q13 Autism Spectrum Disorders
Source: PLoS One. 2012 May 2;7(5):e35414. doi: 10.1371/journal.pone.0035414 (PMC3342283; doi:10.1371/journal.pone.0035414)

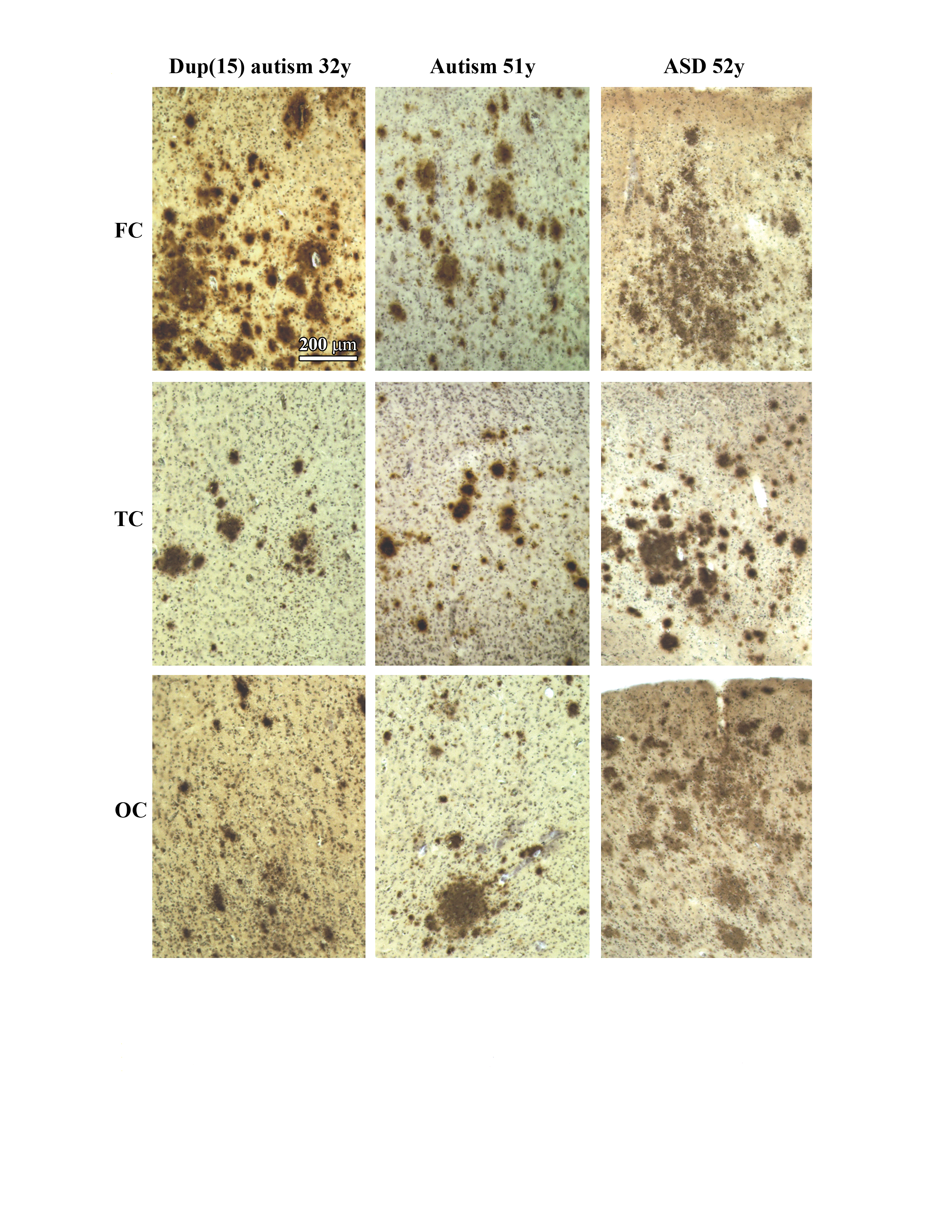

Supplement: Figure S2 — Topography and morphology of neocortical diffuse plaques. Low magnification demonstrates diffuse plaques immunostained with mAb4G8 (17–24 aa) in frontal, temporal and occipital cortex (FC, TC and OC, respectively) in the brain of a 39-year-old female diagnosed with dup(15) autism, a 51-year-old autistic male, and a 52-year-old subject with atypical autism. (TIF) [file pone.0035414.s002.tif]
